# The mitochondrial single-stranded DNA binding protein is essential for initiation of mtDNA replication

**DOI:** 10.1126/sciadv.abf8631

**Published:** 2021-07-02

**Authors:** Min Jiang, Xie Xie, Xuefeng Zhu, Shan Jiang, Dusanka Milenkovic, Jelena Misic, Yonghong Shi, Nirwan Tandukar, Xinping Li, Ilian Atanassov, Louise Jenninger, Emily Hoberg, Sara Albarran-Gutierrez, Zsolt Szilagyi, Bertil Macao, Stefan J. Siira, Valerio Carelli, Jack D. Griffith, Claes M. Gustafsson, Thomas J. Nicholls, Aleksandra Filipovska, Nils-Göran Larsson, Maria Falkenberg

**Affiliations:** 1 Key Laboratory of Growth Regulation and Translational Research of Zhejiang Province, School of Life Sciences, Westlake University, Hangzhou, Zhejiang 310024, China.; 2 Department of Mitochondrial Biology, Max Planck Institute for Biology of Ageing, 50931 Cologne, Germany.; 3 Department of Medical Biochemistry and Cell Biology, University of Gothenburg, PO Box 440, Gothenburg 405 30, Sweden.; 4 Department of Medical Biochemistry and Biophysics, Karolinska Institutet, Stockholm 17177, Sweden.; 5 Lineberger Comprehensive Cancer Center, Department of Microbiology and Immunology, University of North Carolina, Chapel Hill, NC 27514, USA.; 6 Proteomics Core Facility, Max Planck Institute for Biology of Ageing, 50931 Cologne, Germany.; 7 Harry Perkins Institute of Medical Research and ARC Centre of Excellence in Synthetic Biology, Nedlands, WA 6009, Australia.; 8 Telethon Kids Institute, Northern Entrance, Perth Children’s Hospital, 15 Hospital Avenue, Nedlands, WA, Australia.; 9 Department of Biomedical and Neuromotor Sciences (DIBINEM), University of Bologna, Bologna, Italy.; 10 IRCCS Istituto delle Scienze Neurologiche di Bologna, Programma di Neurogenetica, Bologna, Italy.; 11 Wellcome Centre for Mitochondrial Research, Biosciences Institute, The Medical School, Newcastle University, Newcastle upon Tyne NE2 4HH, UK.

## Abstract

We report a role for the mitochondrial single-stranded DNA binding protein (mtSSB) in regulating mitochondrial DNA (mtDNA) replication initiation in mammalian mitochondria. Transcription from the light-strand promoter (LSP) is required both for gene expression and for generating the RNA primers needed for initiation of mtDNA synthesis. In the absence of mtSSB, transcription from LSP is strongly up-regulated, but no replication primers are formed. Using deep sequencing in a mouse knockout model and biochemical reconstitution experiments with pure proteins, we find that mtSSB is necessary to restrict transcription initiation to optimize RNA primer formation at both origins of mtDNA replication. Last, we show that human pathological versions of mtSSB causing severe mitochondrial disease cannot efficiently support primer formation and initiation of mtDNA replication.

## Teaser

The mitochondrial single-stranded DNA binding protein is required for initiation of DNA synthesis in mammalian cells.

## INTRODUCTION

The mammalian mitochondrial genome [mitochondrial DNA (mtDNA)] is essential for cellular energy conversion, encoding 13 of the key subunits of the oxidative phosphorylation system (OXPHOS) as well as tRNA and ribosomal RNA (rRNA) molecules required for mitochondrial translation. All other mitochondrial proteins are nuclear-encoded, including most of the OXPHOS subunits and all factors required for mtDNA maintenance and expression. The mtDNA copy number varies depending on tissue energy requirements, ranging from hundreds to many thousands per cell (*1*). Maintaining proper mtDNA levels is critical for normal development and health, and mtDNA replication occurs independently of the cell cycle (*2*). Defects in mtDNA maintenance cause a heterogeneous group of mitochondrial disorders, characterized at the molecular level by mtDNA depletion or mtDNA deletions, which lead to OXPHOS deficiency in affected tissues (*3*). The underlying causes for many of these disorders are mutations in the core subunits of the mtDNA replication machinery, including DNA polymerase γ (POLγ), the replicative DNA helicase TWINKLE, and mitochondrial single-stranded DNA (ssDNA) binding protein (mtSSB) (*3*–*10*).

In human cells, mtDNA is a small [16,569 base pairs (bp)], circular, double-stranded molecule. The two strands are referred to as the heavy and light strand (H- and L-strand, respectively) due to a strand bias in base content. The mtDNA replication machinery is distinct from that in the nucleus, and some of the core components, including POLγ and TWINKLE, are related to replication proteins present in bacteriophages. In contrast, mtSSB resembles *Escherichia\coli* SSB, forming a tetramer composed of four 16-kDa subunits (*11*–*13*). The mtSSB protein stimulates POLγ processivity and the double-stranded DNA (dsDNA) unwinding activity of TWINKLE at the mitochondrial replication fork (*14*, *15*).

Replication is initiated from a specific origin on each strand, O_H_ and O_L_. In 1972, the Vinograd laboratory (*16*) proposed that mtDNA replication occurs through a strand-displacement mode and that mtDNA synthesis is continuous on both strands. Replication is first initiated at O_H_, leading to the formation of the nascent heavy (H) strand. When about two-thirds of the H-strand have been synthesized, the replication machinery reaches O_L_, which becomes activated, and light strand DNA synthesis commences. Synthesis of the two strands proceeds continuously until two complete daughter molecules are formed (*14*, *17*).

Primer formations at the two origins of replication are key steps in mtDNA replication initiation. At both O_H_ and O_L_, primers are synthesized by the mitochondrial RNA polymerase (POLRMT). Initiation of replication at O_H_ depends on primers formed by transcription from the upstream light-strand promoter (LSP), which terminates prematurely to create a stable R loop (*18*–*20*). The mechanisms regulating the switch between primer formation and genomic length transcription at O_H_ are still under investigation (*1*, *21*, *22*). Factors that modulate POLRMT levels or processivity and the stability of the R loop likely play a role in the balance between transcription and replication. For instance, the mitochondrial transcription elongation factor (TEFM) increases POLRMT transcription processivity and prevents R loop formation (*22*–*24*). However, in vivo TEFM depletion has no effect on mtDNA levels, suggesting that other factors are needed to regulate the transcription-replication switch (*22*). This is based on the notion that a regulatory switch should have opposing effects on transcription and replication.

Primer formation at O_L_ takes place via a different mechanism. When the mitochondrial replication machinery reaches O_L_ during H-strand DNA synthesis, the origin is exposed in its single-stranded conformation and folds into a stem-loop structure (*25*, *26*). A poly-dT stretch in the loop region of the folded origin serves as a start site for POLRMT primer synthesis on the parental H-strand. POLRMT is nonprocessive on the ssDNA and only produces primers of ~25 nucleotides (nt) before leaving the template (*27*). At this point, POLγ uses the 3′-end of the RNA primer to initiate L-strand DNA synthesis. In vitro analysis indicates that mtSSB can play an important role in O_L_-dependent initiation by blocking nonspecific primer formation at other sites on the single-stranded, parent H-strand. At O_L_, the stem-loop structure prevents mtSSB binding, leaving the T-stretch in the single-stranded loop region accessible to POLRMT for initiating primer synthesis (*28*). In support of this model, in vivo occupancy analysis has revealed that mtSSB covers the parental H-strand when it is displaced during mtDNA replication (*28*).

The primary sequence and structure of mtSSB are similar to the *E. coli* SSB. Similar to its *E. coli* paralog, mtSSB displays different modes of DNA binding. On longer stretches of ssDNA (>60 nt), all four mtSSB subunits contact and fully wrap ssDNA. On shorter stretches (~30 nt), only two of the subunits in the tetramer are bound to ssDNA. In contrast to *E. coli* SSB, mtSSB does not bind to ssDNA in a cooperative manner. The reason for this difference is not known but may be explained by the absence of an acidic C terminus present in the *E. coli* protein (*29*–*31*). In the present study, we have developed an unbiased cellular screen to identify factors involved in mtDNA maintenance and identified mtSSB as a key regulator of mtDNA replication. We used a combination of mouse knockout analyses and in vitro analyses of naturally occurring, disease-causing mutations to elucidate the molecular role of mtSSB in mtDNA metabolism. We find that mtSSB is essential for the switch from transcription to replication at O_H_ and to prevent nonspecific priming across the genome. In conclusion, we demonstrate that mtSSB has an unexpected additional function in directing RNA primer formation that is distinct from the classical function to protect ssDNA at the replication fork.

## RESULTS

### mtSSB is critically required for mtDNA maintenance

To identify mitochondrial proteins that regulate mtDNA levels, we used small interfering RNA (siRNA) screening in combination with mtDNA content analysis in single cells. We analyzed 1142 genes from MitoCarta2.0, which is a catalog of genes encoding proteins identified to localize within mitochondria (*32*). Knockdown of *SSBP1*, which encodes mtSSB, caused the most marked decrease in mtDNA levels (figs. S1 to S4). This observation indicates not only that mtSSB may serve to protect and stabilize ssDNA during mtDNA replication as previously suggested (*4*–*6*, *28*) but also that it could have a more direct role in controlling mtDNA replication. To investigate this possibility further, we generated and characterized conditional knockout mice lacking mtSSB and performed a series of biochemical experiments with mutant forms of mtSSB found in patients with mitochondrial disease.

### mtSSB is essential for initiation of mtDNA replication in the mouse

**Fig. 1:**
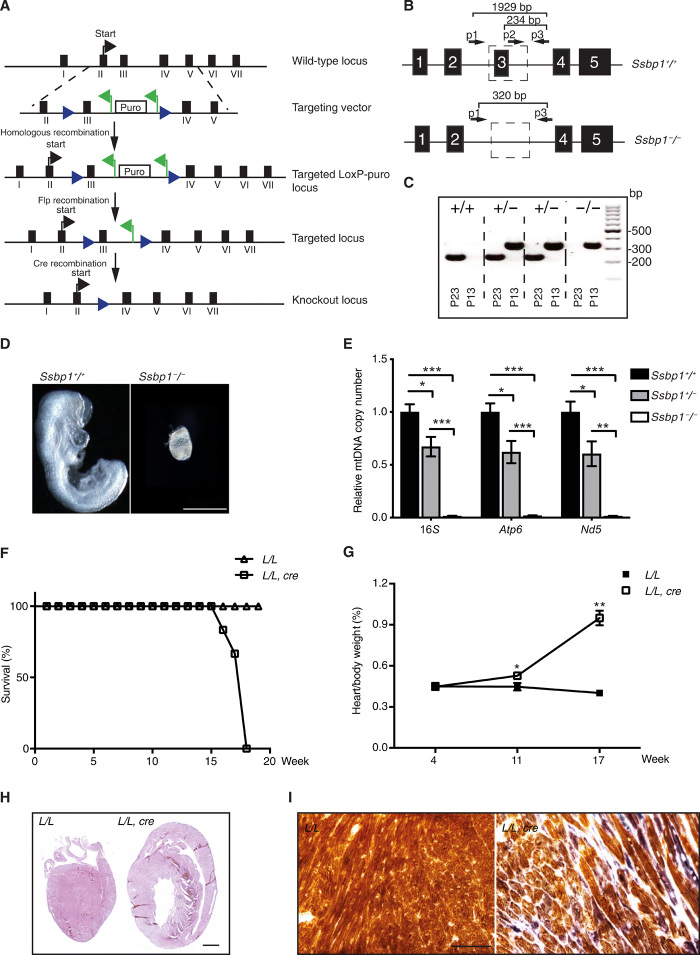
Germline *Ssbp1* knockout leads to embryonic lethality, whereas knockout in the heart and skeletal muscle leads to cardiomyopathy. (**A**) Targeting strategy for disruption of the *Ssbp1* gene. Blue arrowhead, *loxp* sequence; green arrowhead, *frt* sequence. (**B**) Three polymerase chain reaction (PCR) primers designed for detecting *Ssbp1* exon 3. (**C**) Genotyping of *Ssbp1^+/+^*, *Ssbp1^+/−^*, and *Ssbp1^−/−^* tissues by indicated primers. (**D**) Morphology of *Ssbp1^+/+^* and *Ssbp1^−/−^* embryos at E8.5. Scale bar, 0.5 mm. (**E**) Relative mtDNA levels in *Ssbp1^+/+^*, *Ssbp1^+/−^*, and *Ssbp1^−/−^* embryos assessed with real-time quantitative PCR (qPCR). Data are represented as means ± SEM; *P < 0.05, **P < 0.01, and ***P < 0.001. (**F**) Survival curve of *Ssbp1* conditional knockout mice (*L/L, cre*) and controls (*L/L*). (**G**) Heart-to-body weight ratio of *L/L, cre* and *L/L*. (**H** and **I**) Hematoxylin and eosin staining and COX/SDH staining of heart tissues from 17-week-old *L/L, cre* and *L/L*. Scale bars, 1 mm (H) and 100 μm (I).

We established a conditional knockout allele for *Ssbp1* by targeting exon 3 in mice (Fig. 1, A to C). Heterozygous knockout (*Ssbp1^+/−^*) mice were obtained by crossing *Ssbp1^loxP^* mice with β*-actin-cre* mice. Intercrossing of *Ssbp1^+/−^* mice produced no viable homozygous knockout mice (*Ssbp1^−/−^*) after genotyping of a total of 95 offspring from 15 litters [*Ssbp1^+/+^*, *n* = 30 (31.6%); *Ssbp1^+/−^*, *n* = 65 (68.4%); and *Ssbp1^−/−^*, *n* = 0 (0%)]. We then dissected and analyzed embryos at embryonic day 8.5 (E8.5; *n* = 40) and found that *Ssbp1^−/−^* embryos (*n* = 12) had a mutant appearance, whereas the remaining embryos (*Ssbp1^+/−^*, *n* = 18 and *Ssbp1^+/+^*, *n* = 10) appeared normal (Fig. 1D). We observed a marked decrease in mtDNA levels in heterozygous *Ssbp1^+/−^* embryos and loss of mtDNA in homozygous *Ssbp1^−/−^* embryos (Fig. 1E). We thus conclude that *Ssbp1* is essential for embryonic development and that its loss causes embryonic lethality at ~E8.5.

To further study the function of mtSSB, we generated tissue-specific knockout mice by breeding *Ssbp1^loxP/loxP^* mice with transgenic mice expressing Cre recombinase in cardiomyocytes and skeletal muscle under the control of the muscle creatinine kinase promoter (*Ckmm-cre*), which is active from midgestation (*33*). The resulting conditional knockout mice (*Ssbp1*^loxP/loxP^, *+/Ckmm-cre*) had a reduced life span and died at an age of ~18 weeks (Fig. 1F). We analyzed mice at three different ages, 4 to 5, 11, and 17 weeks, and found a progressive increase in the heart-to-body weight ratio (Fig. 1G) and morphological signs of cardiomyopathy in knockout mice at 17 weeks of age (Fig. 1H). A combined cytochrome c oxidase/succinate dehydrogenase activity (COX/SDH) staining of hearts from 17-week-old mice showed a marked respiratory chain deficiency (Fig. 1I).

**Fig. 2:**
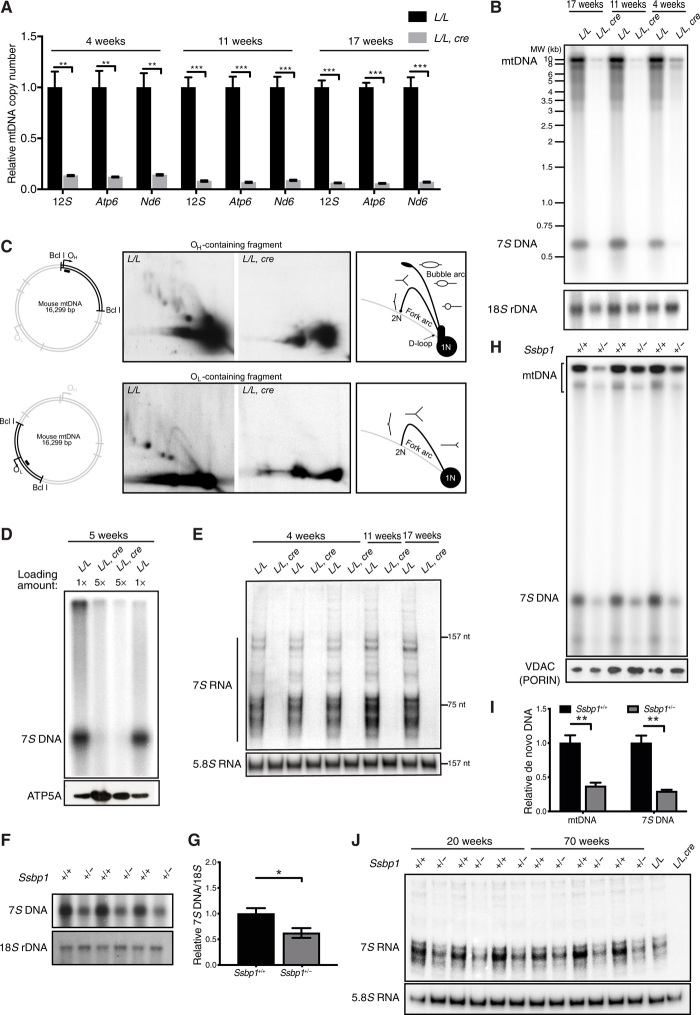
Decreased steady-state levels of mtDNA and abolished mtDNA replication in tissue-specific *Ssbp1* knockout mice. (**A**) Steady-state levels of mtDNA in control (*L/L)* and knockout (*L/L, cre)* mice as determined by qPCR on total heart DNA (*n* > 3 mice per time point). (**B**) Steady-state levels of 7*S* DNA as determined by Southern blotting (*n* ≥ 3 mice per time point). (**C**) Representative mtDNA replication pattern in isolated heart mitochondria at the age of 5 weeks as determined by 2D agarose gel electrophoresis. Restriction enzyme sites are indicated. The black bars indicate the probes used for O_H_ and O_L_. (**D**) De novo synthesized 7*S* DNA from isolated heart mitochondria (*n* = 3). MW, molecular weight; rDNA, ribosomal DNA. (**E**) 7*S* RNA as detected by Northern blotting of heart RNA. 5.8*S* RNA (157 nt) and *tRNA-L1* (75 nt) were used as size markers as indicated. (**F**) Steady-state levels of 7*S* DNA in hearts of *Ssbp1^+/−^* and control mice at 20 weeks of age. (**G**) Quantification of the experimental data displayed in (F) (*n* = 9). (**H**) De novo synthesis of 7*S* DNA and mtDNA in *Ssbp1^+/−^* and control mice. (**I**) Quantification of the experimental data displayed in (H) (*n* = 3). (**J**) Steady-state levels of 7*S* RNA in hearts of *Ssbp1^+/−^* and control mice. RNAs isolated from heart-specific, 4-week-old *Ssbp1* knockout mice (*L/L, cre*) and controls (*L/L*) were also included. All analyzed data are represented as means ± SEM; *P < 0.05, **P < 0.01, and ***P < 0.001.

Analysis of mtDNA levels by real-time quantitative polymerase chain reaction (qPCR) in hearts lacking mtSSB revealed a marked decrease at 4 to 5 weeks of age (13.2% of wild-type levels), with a further reduction at 11 and 17 weeks of age (7.9 and 6.3% of wild-type levels, respectively) (Fig. 2, A and B). We also observed loss of 7*S* DNA (Fig. 2B), which is the short nascent DNA molecule formed during the initiation mtDNA replication at O_H_. To directly assess mtDNA replication, we used two-dimensional (2D) agarose gel electrophoresis and monitored mtDNA replication patterns in 5-week-old mice. In the absence of mtSSB, no replication intermediates were observed (Fig. 2C), indicating that the drop in mtDNA copy number is not the result of incomplete or stalled replication but rather is due to a complete loss of DNA synthesis. Analysis of de novo DNA synthesis in isolated knockout heart mitochondria in 5-week-old mice indeed revealed a marked decrease in 7*S* DNA synthesis and a loss of full-length mtDNA synthesis (Fig. 2D). In support of our results, others have demonstrated that depletion of mtSSB in HeLa cells causes a severe reduction of 7*S* DNA levels (*34*). We also analyzed steady-state levels of 7*S* RNAs, which are short, promoter-proximal, noncoding transcripts formed by premature transcription termination at multiple sites downstream of LSP. In contrast to 7*S* RNA in human cells, the 7*S* RNAs in mice have heterogenous sizes (*22*). The function of 7*S* RNAs is not known, but it has been suggested that these transcripts regulate mtDNA transcription (*35*, *36*). We observed a severe reduction of 7*S* RNA in *Ssbp1* knockout hearts at different ages (Fig. 2E). Proteomic analysis of knockout heart tissue confirmed the absence of mtSSB and a down-regulation of mitoribosomal and OXPHOS protein subunits (fig. S5A). Consistently, the assembly of the respiratory chain complexes that include mtDNA-encoded subunits was affected at both 6 and 17 weeks of age (fig. S5B) because of loss of mtDNA expression caused by the low mtDNA levels (Fig. 2, A and B). In agreement with the drop in mtDNA levels, we observed a decrease in the levels of TFAM (Transcription Factor A, Mitochondrial) (fig. S5A), which is the main protein component of mitochondrial nucleoids (*37*–*39*).

Given the marked effects observed in knockout tissues, we also addressed the effects of a partial reduction of mtSSB levels. Heterozygous knockout (*Ssbp1^+/−^*) mice appeared healthy at 20 and 70 weeks of age and had normal mtDNA and mitochondrial RNA levels (fig. S6, A to E), in contrast with the decreased mtDNA levels observed in *Ssbp1^+/−^* embryos (Fig. 1E). However, adult *Ssbp1^+/−^* mice had a marked reduction in both 7*S* DNA and 7*S* RNA levels (Fig. 2, F to J) despite maintaining normal mtDNA levels (fig. S6D). Analysis of de novo DNA synthesis showed decreased 7*S* DNA and full-length DNA synthesis (Fig. 2, H and I). The presence of normal mtDNA levels in *Ssbp1^+/−^* mice is likely explained by increased stability of mtDNA to compensate for the reduced de novo formation.

In summary, analysis of mtDNA replication patterns in both conditional homozygous knockout and heterozygous *Ssbp1* knockout mice demonstrates a critical role for mtSSB in mtDNA maintenance. Unexpectedly, these results show that mtSSB, besides its role in stabilization of mtDNA during replication, also has a critical role in controlling mtDNA replication initiation.

### Depletion of mtSSB causes an up-regulation of LSP transcription

**Fig. 3:**
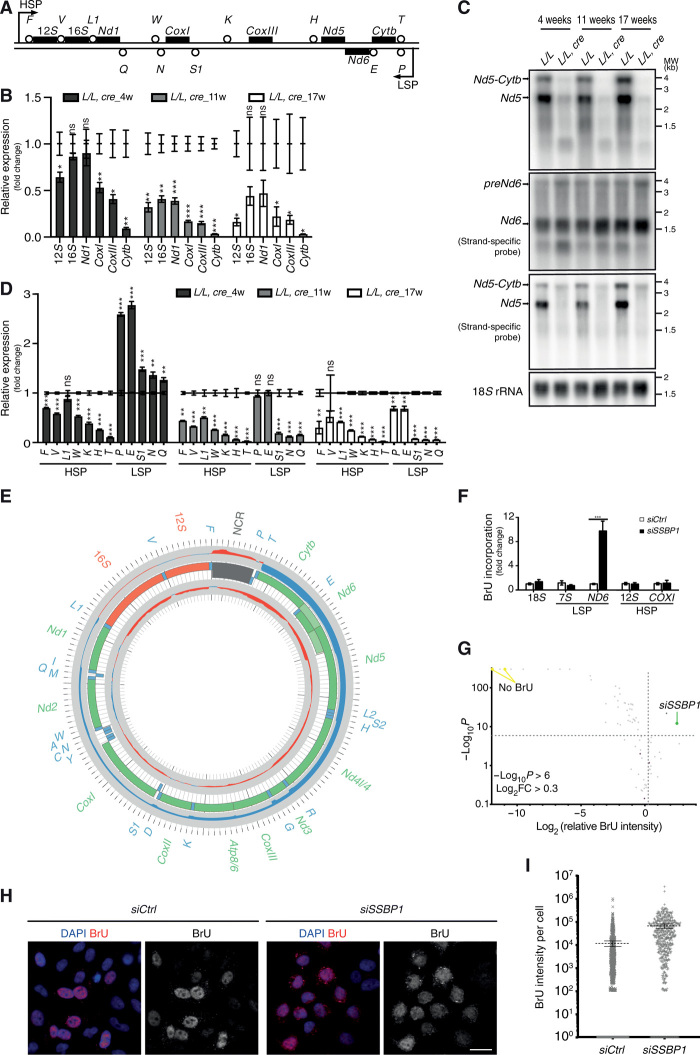
Steady-state levels mitochondrial mRNAs and mitochondrial tRNAs in tissue-specific *Ssbp1* knockout mice. (**A**) Schematic representation of transcripts from HSP and LSP. (**B**) Steady-state levels of H-strand mitochondrial mRNAs in hearts of *Ssbp1* knockout mice in comparison with controls as detected by reverse transcription PCR (RT-PCR). ns, not significant. (**C**) *Nd5* and *Nd6* mRNA levels detected by Northern blots of heart RNA. (**D**) Quantification of tRNAs in hearts in comparison with controls as detected by Northern blots (*n* ≥ 3 mice per time point and group). Means ± SEM; *P < 0.05, **P < 0.01, and ***P < 0.001. (**E**) Changes in the mitochondrial transcriptome from hearts at 5 weeks of age as determined by RNA-seq coverage on the heavy (outer track) and light (inner track) strands (*n* = 3 mice per group). Increases in red and decreases in blue [log_2_(RPM_KO_/RPM_WT_)]. The central track is mtDNA; rRNAs (orange), mRNAs (green), tRNAs (blue), and the noncoding region (NCR; gray) are indicated. (**F**) Quantification of immunoprecipitated BrU-labeled RNA in *siCtrl*- and *siSSBP1*-treated HeLa cells as determined by strand-specific RT-qPCR. Means ± SD (*n* = 3); ***P < 0.001 with unpaired two-tailed Student’s *t* test. (**G**) Volcano plot of BrU incorporation in RNA from 70 knockdown cell lines with decreased mtDNA levels. Each point represents a gene. The BrU incorporation (*x* axis) is plotted against statistical significance (*y* axis). Dashed lines outline genes that significantly increase mitochondrial BrU incorporation upon knockdown. Untreated control samples or samples treated with scramble siRNA are in purple. (**H**) Representative ScanR images of BrU staining in HeLa cells treated with control or *SSBP1* siRNAs. Scale bar, 20 μm. (**I**) Vertical scatter plots showing BrU fluorescence intensity per cell as detected by ScanR. Each dot represents an individual cell. *siCtrl*, *n* = 947; *siSSBP1*, *n* = 574. Lines highlight means ± 95% confidence interval.

To better understand the events leading to the lack of initiation of mtDNA replication in the absence of mtSSB, we investigated the effects on mtDNA transcription. Transcription from LSP forms the primers necessary for initiation of H-strand mtDNA replication at O_H_ and is necessary for expression of the genes located on the L-strand of mtDNA. When we monitored global changes in mitochondrial transcription in knockout heart tissue using real-time qPCR, we found a profound progressive reduction in steady-state levels of transcripts produced from the heavy-strand promoter (HSP) (Fig. 3, A and B) consistent with the observed mtDNA depletion. However, there was an unexpected increase in the levels of *Nd6* transcripts produced from LSP despite the marked drop in mtDNA levels in the knockouts from the age of 4 to 5 weeks (Fig. 3C). A similar effect was seen for levels of tRNAs encoded by the L-strand at the age of 4 to 5 weeks (Fig. 3D and fig. S7). To further validate this unexpected effect on L-strand transcript levels in the absence of mtSSB, we performed genome-wide transcriptomic analysis [RNA sequencing (RNA-seq)] with RNA isolated from the hearts of mice at ~5 weeks of age, which revealed markedly increased steady-state levels of transcripts generated from the LSP (Fig. 3E). The increase in steady-state L-strand transcript levels was most pronounced proximal to LSP and then gradually decreased distally (Fig. 3E and fig. S8, A and B).

To assess how mtSSB affects de novo mtDNA transcription, we depleted mtSSB in HeLa cells in combination with bromouridine (BrU) labeling of newly synthesized transcripts. A specific anti-BrU antibody was used to pull down nascent transcripts, followed by strand-specific reverse transcription and qPCR quantification. We observed a marked increase in de novo formation of the *ND6* transcript synthesized from the L-strand, whereas the levels of de novo 12*S* and *COXI* transcripts synthesized from the H-strand were similar to those observed in the controls (Fig. 3F).

To investigate whether the observed increase in transcription from LSP was a general compensatory response to mtDNA depletion, we analyzed de novo transcription alterations in cells with depletion of the 70 genes that caused mtDNA depletion in the fluorescence in situ hybridization (FISH) screen (Fig. 3, G and H). Only two of these 70 genes (*SSBP1* and *PDK4*) caused a notable increase in BrU incorporation after siRNA knockdown. *SSBP1* had the most pronounced effect (Fig. 3G and table S1). Loss of mtSSB caused both a marked decrease in mtDNA levels [log_2_ fold change (FC) = −1.258 and −log*P* = 177.309; fig. S1, G and H) and an increase in de novo transcription (log_2_FC = 2.503 and −log*P* = 12.11; Fig. 3, H and I). We did not investigate *PDK4* further, but its effect on de novo transcription could be secondary to its role in regulation of lipid and glucose metabolism (*40*).

In summary, our results show that loss of mtSSB affects L-strand transcription. In the absence of mtSSB, there is a marked increase in transcription initiation at LSP, which results in increased steady-state levels of mitochondrial transcripts encoded on the L-strand. The increase in LSP transcription and decrease in mtDNA replication initiation were specific to loss of mtSSB and were not seen when the expression of other mtDNA maintenance factors was reduced.

### Depletion of mtSSB causes aberrant transcription initiation

**Fig. 4:**
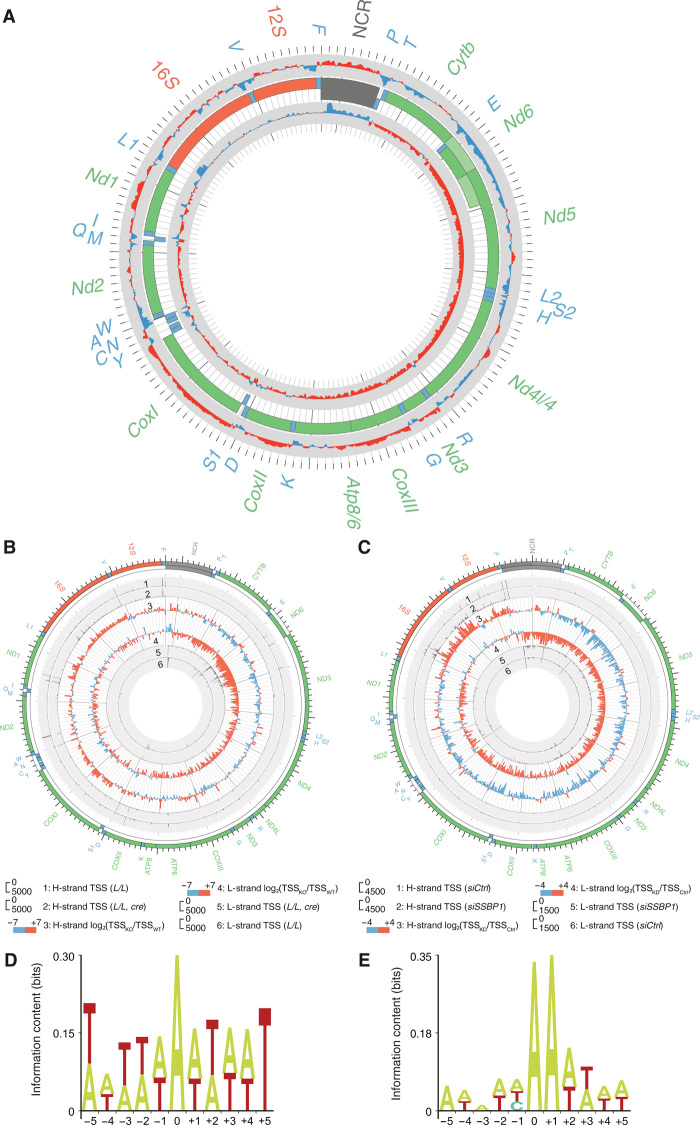
mtSSB inhibits unspecific initiation of RNA synthesis. (**A**) Changes in the mitochondrial transcriptome as determined by small RNA-seq coverage from hearts of 5-week-old mice (*n* = 3 per group). Increases are in red, and decreases are in blue [log_2_(RPM_KO_/RPM_WT_)]. The central track is mtDNA; rRNAs (orange), mRNAs (green), tRNAs (blue), and the NCR (gray) are indicated. (**B**) Changes in mitochondrial TSSs as determined by Cappable-seq of isolated heart mitochondria (*n* = 3 mice per group). The mtDNA is displayed in the outer track. Concentric circles from outermost to innermost show control H-strand TSS (1), knockout H-strand TSS (2), H-strand log_2_(TSS_KO_/TSS_WT_) (3), L-strand log_2_(TSS_KO_/TSS_WT_) (4), knockout L-strand TSS (5), and control L-strand TSS (6). (**C**) Changes in mitochondrial TSSs as determined by Cappable-seq of RNA from control (*siCtrl*) and knockdown (*siSSBP1*) HeLa cells. Two independent biological repeats were performed, and the mean value of changes is presented. The mtDNA is displayed in the outer track. Concentric circles from outermost to innermost show control H-strand TSS (1), knockdown H-strand TSS (2), H-strand log_2_(TSS_KD_/ TSS_Ctrl_) (3), L-strand log_2_(TSS_KD_/ TSS_Ctrl_) (4), knockdown L-strand TSS (5), and control L-strand TSS (6). (**D**) Sequence logo of the nucleotide bias from −5 to +5 positions of nonspecific TSSs from tissue-specific *Ssbp1* knockout mice. (**E**) Sequence logo of the nucleotide bias from −5 to +5 positions of nonspecific TSSs in *SSBP1*-depleted HeLa cells.

To obtain an in-depth analysis of the transcriptional effects in vivo, we sequenced short RNA molecules isolated from heart mitochondria of tissue-specific *Ssbp1* knockout mice and found that loss of mtSSB caused a massive increase of short transcripts, distributed along the mtDNA genome (Fig. 4A and fig. S8, C and D). We proceeded to analyze de novo transcription initiation events using Cappable-seq that identifies 5′-triphosphorylated RNA molecules (*41*). Loss of mtSSB caused a strong increase in nonspecific transcription start sites (TSSs) along the mtDNA genome (Fig. 4B). The total number of capped transcripts originating from nonpromoter sites increased from 35.69 to 65.13% in *Ssbp1* knockout heart tissue. In agreement with the nucleotide specificity demonstrated for POLRMT in vitro, the vast majority of the unspecific transcripts identified contained adenosine triphosphate (ATP) at the 5′-end. Therefore, the data demonstrate that the loss of mtSSB causes transcription initiation from a range of cryptic start sites distributed throughout mtDNA (Fig. 4D). We repeated our Cappable-seq analysis using HeLa cells and found that depletion of *SSBP1* caused an effect similar to the one observed in mouse *Ssbp1* knockout heart (Fig. 4, C and E). Knockdown of *SSBP1* did not affect the patterns of transcription initiation at HSP1 and LSP, and no transcription initiation was observed from the previously proposed HSP2 promoter (fig. S9) (*1*). In summary, our data demonstrate that mtSSB is required to prevent nonspecific RNA synthesis initiation events and acts to restrict primer formation to specific origins of replication.

### mtSSB does not directly affect LSP activity in vitro

**Fig. 5:**
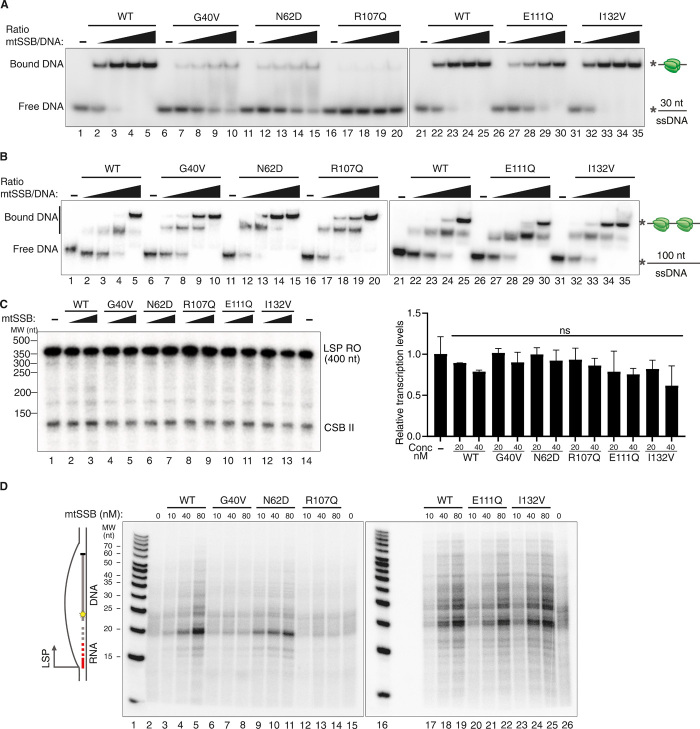
Mutant forms of mtSSB do not support DNA replication initiation at O_H_. (**A**) mtSSB binding to ssDNA oligonucleotides as monitored by EMSA using an ssDNA-fragment. Increasing mtSSB concentrations (0, 1.7, 3.5, 7, and 14 nM, calculated as an mtSSB tetramer) were incubated with 1.7 nM γ-[^32^P] ATP 5′-end labeled DNA (30 nt) for 10 min at room temperature. (**B**) EMSA as described in (A) performed with longer ssDNA oligonucleotides (100 nt). (**C**) In vitro transcription assays with 20 and 40 nM wild-type (WT) or mutated mtSSB. Right: Quantification of run-off products relative to reactions without mtSSB. No significant differences were found with an unpaired two-tailed Student’s *t* test. Means ± SD (*n* = 6 for assay without protein and *n* = 3 for assays with wild-type or mutated mtSSB). (**D**) In vitro replication-initiation assay. Where indicated, wild-type or mutant mtSSB was added. In the experiment presented, only newly synthesized DNA is labeled. Please note that POLγ cannot initiate DNA synthesis de novo but requires an RNA primer. RNA primers not used by POLγ are not visualized in this experiment. For experimental details, please see Materials and Methods. The red line represents RNA primer, and the gray line represents synthesized DNA. The dashed line represents non–size-specific transitions from RNA to DNA.

Mutant forms of mtSSB can cause mtDNA depletion and mitochondrial disease in patients (*4*–*7*). We speculated that these mutant forms of mtSSB could provide interesting insights into the function of the protein during initiation of mtDNA replication. We thus expressed and purified the G40V, N62D, R107Q, E111Q, and I132V mutant forms of mtSSB to near homogeneity. As discussed in Introduction, mtSSB displays different binding modes depending on the ssDNA length (*30*). We analyzed binding of mtSSB to ssDNA of two different lengths (30 and 100 nt). Three mtSSB variants (G40V, N62D, and R107Q) displayed decreased binding to a shorter 30-nt ssDNA fragment in electromobility shift assays (EMSAs). When the experiment was repeated with a longer 100-nt ssDNA fragment, all mutants displayed a binding pattern similar to that seen with wild-type mtSSB (Fig. 5, A and B). Our results suggest that the disease-causing mutations specifically affect the 30-nt binding mode but not the 60-nt binding mode of mtSSB. Since the loss of mtSSB caused increased levels of LSP transcription in vivo, we monitored whether mtSSB could directly affect LSP transcription in a reconstituted system with purified mitochondrial transcription factors (*42*). We observed robust transcription initiation from a DNA fragment containing LSP, and addition of wild-type or mutant variants of mtSSB did not significantly affect this (Fig. 5C). The marked increase in LSP transcripts seen in the absence of mtSSB in vivo is therefore not due to a direct effect of mtSSB on transcription initiation.

### Mutant forms of mtSSB impair initiation of replication at O_H_

We analyzed effects of mtSSB on DNA replication initiation at O_H_. This process can be reconstituted in vitro by combining the mitochondrial transcription machinery with ribonuclease H1 (RNase H1) and POLγ (*43*). Transcription initiated at LSP on a circular, supercoiled plasmid template leads to the formation of a stable R loop in vitro. The R loop is processed by RNase H1, allowing POLγ to use the cleaved RNA molecules as primers for initiation of DNA synthesis. Newly synthesized DNA was labeled by incorporation of α-[^32^P] 3′-deoxythymidine 5′-triphosphate (dTTP), and the reactions were performed in the presence of 2′,3′-dideoxycytidine 5’-triphosphate (ddCTP) to terminate DNA synthesis and form shorter, better defined products. The replication products formed contained an RNA primer linked to radioactively labeled, nascent DNA. RNA-to-DNA transitions take place at a number of different places downstream of LSP, creating a smear of short replication products on the gel. In the absence of mtSSB, we observed a weak reaction (Fig. 5D, lanes 2, 15, and 26). Consistent with previous results (*43*), the addition of mtSSB stimulated replication initiation (Fig. 5D, lanes 3 to 5 and 17 to 19). Three mtSSB mutants (G40V, N62D, and R107Q) caused reduced levels of replication initiation, whereas two variants (E111Q and I132V) supported replication initiation at levels similar to those seen with wild-type mtSSB (Fig. 5D). The mutants that failed to stimulate replication initiation were those that also displayed reduced binding to a short, 30-nt ssDNA template in EMSA (Fig. 5A).

### Mutant forms of mtSSB support replication elongation but impair initiation at O_L_

**Fig. 6:**
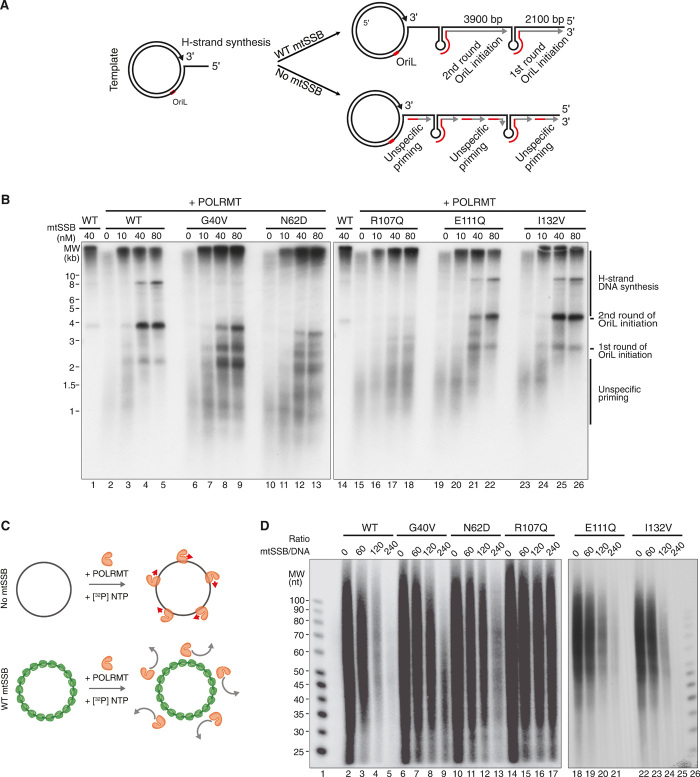
Mutant forms of mtSSB impair replication initiation at O_L_. (**A**) Schematic illustrations of the in vitro replication assay using a dsDNA template. The template contains the O_L_ sequence, allowing for replication-dependent initiation of L-strand DNA synthesis (red). Lack of mtSSB or mutated forms of mtSSB will cause nonspecific DNA synthesis from sequences outside the O_L_ region. (**B**) In vitro replication assay using a dsDNA template containing O_L_ (4 kb) using the indicated mtSSB concentrations. (**C**) Schematic illustration of the nonspecific transcription assay with a random ssDNA template. POLRMT (orange) catalyzes RNA synthesis (red arrows) nonspecifically in the absence of mtSSB (top), and mtSSB (green) inhibits RNA synthesis (bottom). (**D**) Transcription assay on a random circular ssDNA template (7200 nt) showing unspecific transcription patterns depending on the mtSSB/ssDNA ratio.

Next, we investigated whether mutant mtSSB versions could stimulate replication elongation and direct initiation from the second origin of replication, O_L_. We used a template with a preformed replication fork for loading the replication machinery (Fig. 6A, top). The template contained the O_L_ sequence, which controls replication-dependent initiation of lagging-strand (L-strand) DNA synthesis. As previously reported (*28*), wild-type mtSSB stimulated leading-strand (H-strand) DNA synthesis (Fig. 6B). In the absence of mtSSB, we observed abundant nonspecific initiation of mtDNA synthesis from sequences outside of the O_L_ region. The addition of mtSSB blocked unspecific replication initiation events and stimulated O_L_-dependent initiation of lagging-strand replication (Fig. 6, A and B).

Whereas all of the disease-causing mutant versions of mtSSB stimulated leading-strand mtDNA synthesis, only I132V could direct O_L_-specific initiation. The lagging-strand replication initiation pattern observed for R107Q was similar to that seen in the absence of mtSSB, while G40V, N62D, and E111Q could only partially direct initiation to O_L_ (Fig. 6, A and B).

In agreement with this observation, G40V, N62D, R107Q, and E111Q all failed to efficiently block unspecific replication initiation in vitro on an ssDNA template (Fig. 6, C and D). Again, the most pronounced effect was seen with R107Q. The severe effect of R107Q is explained by a significantly higher off rate from ssDNA than wild-type mtSSB (fig. S10A). However, if cross-linked, then R107Q displays a pattern of ssDNA organization similar to the wild-type protein, as judged by electron microscopy (fig. S10B).

In summary, mutant versions of mtSSB that cause mtDNA depletion in affected patients do not affect replication elongation in vitro. In agreement with the findings in *Ssbp1^−/−^* heart tissue reported above, these mutations instead impair initiation of replication from O_H_ and/or O_L_. In our analysis, I132V was the only mtSSB variant that did not affect replication initiation from either of the two origins. This specific mutation causes a reduction of mtSSB levels in vivo (*4*). The I132V mutant phenotype is therefore most likely a consequence of insufficient mtSSB levels rather than impaired molecular function.

## DISCUSSION

Here, we demonstrate that mtSSB is crucial for maintaining adequate levels of mtDNA. The down-regulation of mtSSB caused the most profound loss of mtDNA in a knockdown screen of 1142 mitochondrial proteins in the MitoCarta2.0 database. In agreement with these findings, *Ssbp1* is essential for embryonic development in mice and is required to establish normal mtDNA levels during development. Previous work has identified a set of pathogenic mutations in the *SSBP1* gene that cause severe phenotypes in affected individuals, including optic atrophy and kidney insufficiency (*4*–*7*). At the molecular level, these mutations are associated with mtDNA depletion. Confirming the findings of the mouse knockout model, the patient mutations impair primer formation and initiation of mtDNA replication in vitro, both at O_H_ and O_L_.

Our results establish that the profound reduction in mtDNA copy number caused by loss of mtSSB derives from a failure of replication initiation rather than from defects in replication elongation. The impaired de novo mtDNA synthesis is not accompanied by the appearance of replication intermediates in *Ssbp1* knockout mice. In addition, all of the mutant mtSSB variants causing human disease could efficiently stimulate replication elongation in vitro (Fig. 6B) (*4*). mtSSB is clearly required for both O_H_-dependent initiation of replication and stimulation of fork progression. However, in the absence of mtSSB, replication cannot be initiated from O_H_ and the effects on elongation, therefore, become secondary. In this regard, note that patients carrying pathological mutations in *SSBP1* show mtDNA depletion but not multiple deletions. These deletions are a common feature associated with mutations in other proteins that act at the mitochondrial replication fork, e.g., POLγ, TWINKLE, and mitochondrial genome maintenance exonuclease 1 (MGME1), and are typically accompanied by replication stalling. The data that we present here are thus consistent with a model arguing that replication progression defects are a primary cause of deletion formation.

The primers required for replication initiation at both O_H_ and O_L_ are formed by POLRMT. However, the requirement for mtSSB differs between the two origins. At O_H_, premature termination of RNA synthesis leads to the formation of an R loop that is subsequently processed by RNase H1. The 3′-ends formed are used to prime mtDNA synthesis by POLγ. Previous work has demonstrated that mtSSB can stabilize the R loop (*43*). A similar stabilizing effect has also been observed in other systems, in which SSBs can promote R loop formation by binding to the displaced DNA strand (*44*–*46*). A possible interpretation of our data is that some mutant forms of mtSSB fail to stabilize the R loop. In support of this notion, the same mutant variants that were unable to stimulate O_H_ activation also failed to bind shorter stretches of ssDNA (compare Fig. 5, A and D).

mtSSB depletion caused a marked up-regulation of transcription from LSP, resulting in increased steady-state levels of *Nd6* and LSP-derived tRNAs, especially those expressed from promoter-proximal genes. A similar response is not seen after knockdown of other genes required for mtDNA maintenance and is therefore not a general compensatory effect. The strong up-regulation of transcription from LSP is most likely connected to the inability to switch to primer formation for initiation of mtDNA synthesis at O_H_. We have previously demonstrated that R loop formation causes premature termination of transcription elongation at a conserved sequence box (CSB II) positioned about 120-nt downstream of LSP (*20*, *47*). The increased levels of LSP transcripts seen in the absence of mtSSB could therefore be explained by destabilized R loop formation and increased levels of read-through transcription in this region. The mtSSB protein thus has an important role in controlling the switch between transcription for replication primer formation and near-genome length transcription for gene expression.

Note that the effects observed in *Ssbp1* knockout hearts are distinctly different from those observed following the loss of TEFM. In the latter case, transcription events terminate before reaching the O_H_ region and therefore cannot support primer formation and mtDNA replication. Instead, TEFM acts as a general transcription elongation factor, necessary for both full-length transcription and regulation of replication primer formation (*22*).

Adequate levels of mtSSB are also required to restrict primer formation to O_L_ during the first phases of replication of the displaced DNA strand. The role that we describe here for mtSSB has similarities to what has been described for the *E. coli* SSB protein, which also plays an essential role in directing primer formation. In filamentous bacteriophages, such as M13, the primer required for DNA synthesis of the minus strand is synthesized by the host RNA polymerase (*48*, *49*). However, in the absence of SSB, there is an alternative mechanism that denoted the general priming reaction. Under these circumstances, the *E. coli* primase, together with the DnaB helicase, is able to synthesize multiple short RNA primers distributed along the single-stranded template. When SSB is added, it suppresses the general priming reaction and instead directs the formation of unique primers at origins of replication. This mechanism operates in several phage replication systems, including G4, ϕX174, and M13 (*50*). The mechanism, thus, has important similarities to the one that we describe here for mammalian mtDNA replication. When the mitochondrial primase POLRMT is incubated with a single-stranded template, short RNA primers are formed at multiple positions. During the first phase of strand displacement replication, this leads to the initiation of L-strand DNA synthesis from multiple sites. The addition of mtSSB inhibits these random priming events and ensures origin-specific initiation of primer synthesis at O_L_ followed by L-strand DNA replication. Thus, both mtSSB and its bacterial relative SSB are required to ensure specific initiation at proper origins of replication.

In summary, our results demonstrate that mtSSB is essential for the switch from transcription to replication at O_H_ and for the specific initiation of replication at O_L_. The absence of mtSSB is associated with a failure of replication initiation, as well as unrestricted initiation of RNA synthesis throughout the mitochondrial genome.

## MATERIALS AND METHODS

Key resources and reagents are listed in table S2.

### Mouse models

Generation of germline and conditional *Ssbp1* knockout mice was performed by Taconic Biosciences. The targeting vector has been generated using bacterial artificial chromosome (BAC) clones from the C57BL/6J RPCI-23 BAC library and transfected into the C57BL/6N Tac embryonic stem cell line by TaconicArtemis. For disrupting *Ssbp1* expression, exon 3 of *Ssbp1* was flanked by *loxp* sites with a puromycin resistance (PuroR) cassette as positive selection marker. After crossing with mice harboring *Flp* recombinase, the PuroR cassette was removed, resulting in *Ssbp1^+/loxp^* mice. For germline knockout mice, *Ssbp1^+/loxp^* mice were mated with mice ubiquitously expressing *cre* recombinase under the β-actin promoter to generate heterozygous knockout *Ssbp1^+/−^* mice. For the conditional knockout of mtSSB in the heart and skeletal muscle, *Ssbp1^+/loxp^* were mated with mice expressing *cre* recombinase under *Ckmm* (the muscle creatinine kinase) promoter.

These mice are part of the breeding permit: AZ: 84-02. 04. 2015. A103, approved by the Landesamt für Natur, Umwelt und Verbraucherschutz, Nordrhein-Westfalen, Germany. Dissections were performed under AZ: 84-02. 05. 50. 15. 004. All mice maintaining an inbred C57BL/6N nuclear background were housed in a 12-hour light/dark cycle at 21°C and fed ad libitum on a standard mouse food (ssniff RM-H Low-Phytoestrogen). The health status of the animals is specific pathogen–free according to Federation of the European Laboratory Animal Science Association recommendations. Mice at the age of 4 to 6, 11, and 17 weeks were used for the histological and biochemical analysis.

### Cell lines

HeLa cells were grown at 37°C and 5% CO_2_ in Dulbecco’s modified Eagle’s medium GlutaMAX supplemented with 10% fetal bovine serum. HeLa cells were reverse-transfected with the indicated siRNA (10 nM) using Lipofectamine RNAiMAX. Experiments were performed 72 hours after transfection.

### mtDNA FISH and immunofluorescence

After siRNA transfection, HeLa cells were plated on 12-mm coverslips (for confocal microscopy) or 96-well plates (for ScanR imaging quantitative analysis) for 72 hours. After fixation with 4% formaldehyde for 20 min, cells were permeabilized using 0.5% Triton X-100 for 10 min. Cells were treated with an RNase mix [RNase A (0.1 mg/ml) and RNase H (100 U/ml)] at 37°C for 1 hour. Prehybridization and hybridization were performed in buffer containing 10% dextran sulfate, 2× SSC, 50% formamide, and salmon sperm DNA (1 μg/μl) at 37°C for 1.5 hours after denaturation for 3 min at 91°C (*51*). We designed five primer pairs to amplify the human mtDNA genome, excluding the D loop region. Each primer pair amplify a ~3-kb fragment, and the PCR products were mixed at an equimolar ratio to create the mtDNA probe (*52*). The probe was labeled by nick translation with ATTO488–deoxyuridine triphosphate (dUTP) or ATTO550-dUTP, purified through a DNA purification column, and added to the hybridization buffer at a final concentration of 1 ng/μl. After adding the indicated probes, coverslips were sealed with rubber cement, and 96 plates were sealed with PCR plate sealing film, denatured at 91°C for 3 min, and hybridized at 37°C overnight. Samples were then washed five times in 2× SSC containing 0.1% Tween 20 and 0.1% Triton X-100. For samples requiring colabeling of the mitochondrial network, coverslips were blocked in 2% bovine serum albumin (BSA) and incubated for 1 hour each in rabbit anti-COXIV (1:200) and Alexa Fluor 488 goat anti-rabbit (1:250). Cell nuclei were counterstained with 300 nM 4′,6-diamidino-2-phenylindole (DAPI) before mounting in ProLong Diamond or ScanR imaging.

For BrU labeling, cells were plated on 96-well plates, transfected with the indicated siRNAs, and incubated for 72 hours at 5% CO_2_. After addition of culture medium containing 2 mM freshly prepared BrU, the cells were incubated for 1 hour at 37°C, and immunofluorescence staining was performed as described above using mouse anti–5-bromo-2′-deoxyuridine (BrdU; 1:200; Millipore, JA1599) and Alexa Fluor 555 donkey anti-mouse (1:250).

### High-content FISH imaging screening

HeLa cells were transfected with siRNAs targeting 1142 human genes encoding mitochondrial proteins (Human MitoCarta2.0), followed by incubation in 96-well plates for 72 hours. Scrambled control siRNA-treated cells and cells treated with a serial dilution of ddCTP were included in each plate as internal controls. FISH was performed as described above. The Olympus ScanR microscope station was used to acquire images. After the first round of screening, candidates were selected on the basis of fold changes (|log_2_| >0.3) and *P* values (−log*P* > 6) and screened again. Only verified results were considered as final candidates.

### Microscopy, imaging analysis, and statistics

A Zeiss LSM 880 equipped with an Airyscan detector was used for high-resolution imaging acquisition. The oil objective is a Plan-Apochromat 63×/1.4 numerical aperture (NA) objective. Stack images were acquired in sequential mode with a pixel dwell time of 1.65 μs, a pinhole of 133 μm, a pixel size of 35 nm by 35 nm, and a z-spacing of 160 nm.

For quantitative imaging analysis, nine nonoverlapping images were acquired for each condition using an Olympus ScanR microscope with an UPLSAPO 20×/0.75 NA objective. At least 500 cells were analyzed for each condition, and images were processed using ScanR analysis software. We stained with DAPI, identified nuclei using edge detection, and gated on the basis of area, circularity, and mean intensity. Total mtDNA FISH intensity or BrU immunofluorescence intensity within 30 pixels (12 μm) outside of each nuclear area was scored. The *P* values were calculated using a two-tailed Student’s *t* test, and volcano plots and scatter plots were prepared using GraphPad Prism 8. Gene ontology analysis was performed using the online database PANTHER.

### Histology

Hearts collected from 17-week-old mice were washed with ice-cold phosphate-buffered saline (PBS) buffer, transferred into 1 M KCl in PBS until beating stopped, washed with PBS, and fixed in 4% paraformaldehyde at 4°C for 24 hours. Then, the hearts were processed routinely, embedded in paraffin, and sectioned to 5-μm thickness. Hematoxylin and eosin staining was performed for structural analysis.

### COX/SDH double-labeling enzyme histochemistry

COX/SDH double staining was performed as previously described. Briefly, fresh hearts were collected immediately after dissection and frozen in isopentane chilled with liquid nitrogen, cryosectioned into 7-μm-thick sections, mounted on slides, and briefly left to air dry. Freshly prepared buffer A (0.8 ml of 5 mM 3,3′-diaminobenzidine tetrahydrochloride, 0.2 ml of 500 μM cytochrome c, and 10 μl of catalase) was added to the slides. After incubation for 60 min at 37°C, slides were washed three times with PBS. Thereafter, freshly prepared buffer B [0.8 ml of nitroblue tetrazolium (1.875 mM), 0.1 ml of sodium succinate (1.3 M), 0.1 ml of phenazine methosulfate (2.0 mM), and 10 μl of sodium azide (100 mM)] was applied, and the slides were incubated for 30 min at 37°C. Slides were washed three times with PBS, dehydrated, and mounted for bright-field microscopy.

### mtDNA copy number quantification

Total genomic DNA from HeLa cell pellets was isolated using a Zymo genomic DNA purification kit according to the manufacturer’s instructions. The mtDNA copy number was measured using qPCR with the following reagents: iTaq Universal SYBR Green Supermix and the gene-specific primers (*Cytb* and 18*S*) from Eurofins Genomics. The levels of mtDNA were assessed using primers specific for the mitochondrial gene *Cytb*, whereas primers for nuclear 18*S* rRNA gene were used for loading control. Southern blotting was also used to quantify mtDNA and 7*S* DNA levels. For human cells, total genomic DNA (2 μg) was digested using 20 U of Bam HI at 37°C for 1 hour. For mouse samples, total genomic DNA was digested with SacI-HF at 37°C overnight and preheated at 93°C for 3 min, followed by cooling on ice before loading onto the gel. After electrophoresis in 1% agarose, DNA was depurinated by incubation in 0.25 M HCl for 10 min and incubated in denaturation buffer (0.5 M NaOH and 1.5 M NaCl) twice for 30 min and neutralization buffer [0.5 M tris-HCl (pH 7.4) and 1.5 M NaCl] twice for 30 min. DNA was blotted onto a Hybond N+ nitrocellulose membrane overnight and then cross-linked by exposure to 254-nm ultraviolet, 200 mJ/cm^2^. Radiolabeled dsDNA probes were prepared by random primer labeling of gel-extracted human 7*S* DNA PCR products or mouse 7*S* DNA probe (*53*). Band intensities were quantified using ImageJ software.

Total DNA from heart tissue was isolated using a Gentra Puregene Tissue kit according to the manufacturer’s instructions. Total DNA from E8.5 embryos were denatured in buffer I (25 mM NaOH and 0.2 mM EDTA) at 96°C for 20 min and neutralized using buffer II [40 mM tris (pH 7.8)]. mtDNA copy number was determined using real-time qPCR with the following reagents: TaqMan Universal PCR Master Mix and the TaqMan probes (12*S*, *Atp6*, *Nd6*, and 18*S*). mtDNA levels were assessed using probes against the mitochondrial genes 12*S*, *Atp6*, and *Nd6*; nuclear 18*S* was used as a loading control.

### Mitochondrial mRNA and tRNA quantification with Northern blotting and qPCR

Total RNA from heart tissue was isolated using TRIzol reagent. RNA levels were detected either using real-time qPCR or by Northern blotting. For real-time qPCR, all RNA samples were treated with deoxyribonuclease (DNase) to reduce the potential risk of DNA contamination. TaqMan Universal PCR Master Mix and TaqMan probes (12*S*, 16*S*, *Nd1*, *CoxI*, *CoxIII*, *Cytb*, and 18*S*) from Thermo Fisher Scientific were used. For Northern blotting, strand-specific probes labeled with α-[^32^P] UTP (uridine 5′-triphosphate) specific to *Nd5* and *Nd6* were generated with the Riboprobe System T7 Kit. All probes specific to tRNAs were labeled with γ-[^32^P] ATP using T4 Polynucleotide Kinase. Please note that *Nd6* was analyzed using Northern blotting since this gene overlaps with a long RNA molecule spanning from *Nd5* to *Cytb* on the opposite strand. Band intensities from Northern blotting were quantified using ImageQuant TL 8.1 software.

### De novo replication assays

Freshly isolated heart mitochondria (1 mg) were resuspended in 1 ml of incubation buffer [25 mM sucrose, 75 mM sorbitol, 100 mM KCl, 10 mM K_2_HPO_4_, 0.05 mM EDTA, 5 mM MgCl_2_, 1 mM adenosine 5′-diphosphate, 10 mM glutamate, 2.5 mM malate, and 10 mM tris-HCl (pH 7.4)] supplemented with fatty acid-free BSA (1 mg/ml), 50 μM dTTP, 50 μM dCTP, 50 μM 2′-deoxyguanosine 5′-triphosphate (dGTP), and 20-μCi α-[^32^P] dATP. Incubation was carried out at 37°C for 2 hours on a rotating wheel. After incubation, mitochondria were pelleted at 9000 rpm for 4 min and washed twice with washing buffer. mtDNA was extracted with a Puregene kit, heated at 95°C for 5 min to release 7*S* DNA from mtDNA, separated on a 1% agarose gel, and transferred to nitrocellulose Hybond membrane for exposure to a phosphorimaging screen. Immunoblotting of the Voltage-dependent anion channel (VDAC) protein was used as a loading control and for quantitative normalization.

### 5-BrU–immunoprecipitated strand-specific reverse transcription PCR

Cells plated on 10-cm dishes were transfected with the indicated siRNA for 72 hours and further incubated in culture medium containing 2 mM freshly prepared BrU for 1 hour at 37°C and 5% CO_2_. Total RNA from the cell pellets was extracted using a Quick-RNA Miniprep kit. RNA (50 μg) was denatured for 2 min at 80°C and then incubated with rotation with 2 μl of anti-BrdU (1:100; Millipore, JA1599) in a final volume of 200 μl of immunoprecipitation (IP) buffer [20 mM tris-HCl (pH 7.5), 250 mM NaCl, BSA (1 μg/μl), and RNase inhibitor (40 U/ml)] at 4°C for 1 hour. Suspended anti-mouse immunoglobulin G Dynabeads (20 μl) were added for another 1 hour of incubation. Beads were washed four times with IP buffer, and RNA was eluted with 100 μl of elution buffer (0.1% SDS in RNase-free H_2_O) (*54*). Eluted RNA was cleaned up and concentrated using an RNA cleanup kit. Purified RNA (5 μl) was reverse-transcribed using an iScript Select cDNA synthesis kit using random primers or strand-specific primers against 7*S* RNA, *ND6*, 12*S*, and *COXI*. Random primer-synthesized cDNA was detected with nuclear 18*S* used as a loading control.

### 2D agarose gel electrophoresis

For 2D gels, fresh heart mitochondria were isolated from 5-week-old mice and purified with sucrose gradients. mtDNA was isolated by phenol/chloroform extraction. mtDNA (3 μg) from each sample was digested with Bcl I, precipitated, and loaded onto the 1D gels (0.4% agarose without ethidium bromide). These were run at 27 V for 18 hours at room temperature. The DNA-containing lanes were cut and rotated 90° counterclockwise, and molten 1% agarose containing ethidium bromide (500 ng/ml) was cast around the gel slices. 2D gels were run at constant 260 mA for 6 hours at 4°C. Gels were transferred onto nylon membranes and hybridized with probes detecting either the O_H_-containing fragment (nt 12,034 to nt 16,180) or the O_L_-containing fragment (nt 3102 to nt 7084). Probes were produced by PCR.

### Mitochondrial OXPHOS complex enzyme activity assay

In-gel activity assays were performed as previously described (*55*) except that iodonitrotetrazolium chloride was used instead of nitrotetrazolium blue chloride. Briefly, 75 μg of isolated mitochondria were lysed in 50 μl of solubilization buffer [20 mM tris-HCl (pH 7.4), 0.1 mM EDTA, 50 mM NaCl, and 10% (v/v) glycerol containing 1% (w/v) digitonin] and mixed with loading dye [5% (w/v) Coomassie Brilliant Blue G-250, 150 mM bis-tris, and 500 mM ε-amino-*n*-caproic acid (pH 7.0)]. Blue native–polyacrylamide gel electrophoresis samples were resolved on 3 to 10% gels and further subjected to in-gel activity staining for complexes I, II, and IV.

### Mitochondrial proteome analysis

Sample preparation of ultrapure mitochondria was performed as previously described (*56*) with the following modifications: Desalted peptides were eluted in 40% acetonitrile/0.1% formic acid from the StageTips. For Tandem Mass Tag (TMT) labeling, 4 μg of peptides from each individual sample were resuspended in 9 μl of 100 mM triethylammonium bicarbonate. For mice of three different ages, wild-type and knockout heart samples were distributed in one TMT 10-plex set. Eight micrograms of each TMT 10-plex labeling reagent dissolved in 7 μl of anhydrous acetonitrile was added to each sample and incubated for 1 hour at room temperature. To stop the reaction, 2 μl of 5% hydroxylamine was added to each sample. After 15 min of incubation, 10 samples of each TMT 10-plex set were combined. After drying the samples in a SpeedVac, 100 μl of 0.1% formic acid was added. The 10-plex mixtures were cleaned with C18 StageTips as described before (*57*).

Each of the 10-plex mixtures was fractionated by basic pH reversed-phase high-performance liquid chromatography (LC). We used an UltiMate 3000 Micro-LC from Thermo Fisher Scientific. Peptides were subjected to a Waters ACQUITY UPLC Peptide CSH C18 Column [1.7-μm particles; inside diameter (ID), 1 mm; and length, 150 mm]. Buffers A and B were 10 mM ammonium bicarbonate in 5% acetonitrile (pH 8.0) and 10 mM ammonium bicarbonate in 80% acetonitrile (pH 8.0), respectively. The peptide separation was performed with an 85-min linear gradient from 1 to 45% of buffer B at a flow rate of 30 μl/min. The peptide mixtures were fractionated into 66 fractions and consolidated into 11 fractions. The fractions were dried in a SpeedVac and resuspended with 0.1% formic acid before analysis by LC–tandem mass spectrometry (LC-MS/MS).

### LC-MS/MS analysis

Peptides were separated on a 50-cm, 75-μm-ID Acclaim PepMap rapid separation liquid chromatography (RSLC) analytical column. Buffers A and B were 0.1% formic acid in water and 0.1% formic acid in 80% acetonitrile, respectively. Peptides were separated on a segmented gradient from 6 to 31% buffer B for 120 min and from 31 to 50% buffer B for 10 min at 250 nl/min. Eluting peptides were analyzed on an Orbitrap Fusion Lumos Tribrid mass spectrometer (Thermo Fisher Scientific). Peptide precursor mass/charge ratio (*m*/*z*) measurements were carried out at a resolution of 60,000 in the range of 350 to 1500 *m*/*z*. The automatic gain control (AGC) was set to 1 × 10^6^, and the maximum injection time was set to 100 ms. Peptide fragmentation was performed in the ion trap using 35% normalized collision energy for collision-induced dissociation. The isolation window was 0.7 Da. MS2 spectra were acquired using AGC target of 1 × 10^4^ and 50-ms maximum injection time. The top 10 most intense MS2 fragments were isolated with an isolation window of 1.3 Da using synchronous precursor selection and fragmented using 65% normalized collision energy. The corresponding MS3 scans were acquired in the Orbitrap mass analyzer at a resolution of 50,000. For the MS3 scans, the *m*/*z* range was set from 100 to 500, and the AGC was set to 5 × 10^4^ with a maximum injection time of 86 ms.

### Proteomic data analysis

MaxQuant (*58*) version 1.5.3.8 with integrated Andromeda search engine was used for analyzing the LC-MS/MS raw data (*59*). The raw data were searched against the reviewed and unreviewed sequences of the mouse proteome, UP000000589, from UniProt (downloaded in September 2019). The following parameters were used for data analysis: for “fixed modification,” cysteine carbamidomethylation; for “variable modification,” methionine oxidation and protein N-terminal acetylation; for “digestion” specific with Trypsin/P, maximum missed cleavages is set to 2; for quantification “type,” reporter ion MS3 and 10-plex TMT; the remaining parameters were set as default. TMT reporter correction factors were changed to the values provided by the manufacturer.

TMT reporter intensity data were processed separately for each of the three ages. Proteins with less than 10 valid values were excluded from the analysis. TMT reporter intensities were subjected to variance stabilization normalization using vsn version 3.46.0 (*60*). Differential expression analysis was performed using limma version 3.34.5 (*61*). The differential expression results from the three ages combined and MitoCarta2 annotations were added using the primary gene name and the first of the gene name synonyms of the oldest UniProt identification with the highest number of peptides (*32*). Exploratory data analysis was performed in R version 3.4.3 using the following packages: dplyr version 0.7.6, ggplot2 version 3.0.0, GGally version 1.4.0, FactoMineR version 1.39, and factoextra version 1.0.5.

### Electrophoretic mobility shift assay

To study the DNA binding activity, two different templates were used. Either a short template consisting of a 5′-^32^P–labeled 30-nt oligonucleotide or a longer 100-nt oligonucleotide. Reactions contained 1.7 nM oligonucleotide in 20 mM tris-HCl (pH 7.8), 10 mM MgCl_2_, BSA (0.1 mg/ml), 10 mM dithiothreitol (DTT), 10% glycerol, 2 mM ATP, and indicated amounts of mtSSB in a final volume of 15 μl. The reactions were incubated for 10 min at room temperature before separation on 8% native polyacrylamide gels [0.5× tris-borate EDTA (TBE)] and visualization by autoradiography.

For the competition assays, each time point consisted of 3.5 nM mtSSB, 1.7 nM ^32^P-labeled 100-nt oligonucleotide, 20 mM tris-HCl (pH 7.8), 10 mM MgCl_2_, BSA (0.1 mg/ml), 10 mM DTT, 10% glycerol, and 2 mM ATP in a final volume of 15 μl. The mixture was preincubated at room temperature for 10 min, followed by the addition of 170 nM (100× excess) cold (nonlabeled) 100-nt oligonucleotide. At the indicated time points, the samples were placed on ice until all the points were collected and subsequently separated on 8% native polyacrylamide gels (0.5× TBE) and visualized by autoradiography.

### Electron microscopy

To prepare complexes of mtSSB and ssDNA for electron microscopy, single-stranded M13mp18 DNA (1 μg/ml) was mixed with purified mtSSB wild-type and mutant proteins at ratios from 1:3 to 1:9 (micrograms of DNA:micrograms of mtSSB) in buffer [100 mM NaCl, 10 mM Hepes (pH 7.6), and 1 mM EDTA] on ice for 20 min, followed by fixation with 0.6% glutaraldehyde for 5 min on ice. The samples were applied to thin carbon foils supported by 400 mesh copper screens, dehydrated in an ethanol series, and rotary shadowcast with tungsten. Samples were examined using an FEI T12 transmission electron microscope at 40 kV, and images were recorded using a Gatan Orius camera.

### Rolling circle replication

To study the effects of extensive DNA polymerization (rolling circle replication), we used a template consisting of a 70-mer oligonucleotide hybridized to single-stranded pBluescript SK(+) O_L_, followed by one cycle of polymerization with KOD polymerase to produce an ~4000-bp double-stranded template with a preformed replication fork as described previously (*25*). This rolling circle template (0.4 nM) was added to a reaction mixture (25 μl) containing 25 mM tris-HCl (pH 7.6), 10 mM DTT, 10 mM MgCl_2_, BSA (0.1 mg/ml), 4 mM ATP, 100 μM dATP, 100 μM dTTP, 100 μM dGTP, 25 μM dCTP, 2 μCi of α-[^32^P] dCTP, 150 μM UTP, 150 μM GTP, 150 μM CTP, 4 nM POLRMT, 8 nM TWINKLE (as a hexamer), 10 nM POLγA/B holoenzyme, and the indicated amounts of mtSSB versions. The reaction was incubated at 37°C for 75 min and stopped by the addition of 12.5 μl of alkaline loading buffer [18% (w/v) Ficoll, 300 nM NaOH, 60 mM EDTA (pH 8.0), 0.15% (w/v) bromocresol green, and 0.25% (w/v) xylene cyanol] before loading onto a 0.8% denaturing agarose gel.

### In vitro transcription on ssDNA

For the study of mtSSB and its involvement in primer synthesis, we performed transcription assays using two different methods. For the in vitro transcription on unspecific ssDNA, each 25-μl reaction contained 10 mM tris-HCl (pH 8.0), 20 mM MgCl_2_, 1 mM DTT, BSA (100 μg/ml), 400 μM ATP, 150 μM CTP and GTP, 10 μM UTP, 0.2 μM α-[^32^P] UTP (3000 Ci/mmol), 1.4 nM single-stranded M13mp18 DNA (New England Biolabs), 4 U of RNase inhibitor (Thermo Fisher Scientific), 20 nM POLRMT, and the indicated amounts of each mtSSB version. The reactions were stopped after 30 min at 32°C by adding 200 μl of stop buffer [10 mM tris-HCl (pH 8.0), 0.2 M NaCl, 1 mM EDTA, and glycogen (0.1 mg/ml)]. For the in vitro transcription on LSP-containing templates, the reaction mixture contained 4 nM linearized LSP template (nt 1 to 477 of mtDNA), 20 mM tris-HCl (pH 8.0), 10 mM MgCl_2_, 10 mM DTT, BSA (100 μg/ml), 400 μM ATP, 150 μM CTP, 150 μM GTP, 15 μM UTP, 2 μCi of α-[^32^P] UTP, 4 U of RNase inhibitor, 16 nM POLRMT, 3.2 nM Transcription Factor B2, Mitochondrial (TFB2M), 80 nM TFAM, and the indicated amounts of each mtSSB version. The reactions were stopped after 30 min at 32°C by adding 200 μl of stop buffer as described above. Samples were dissolved in 10 μl of loading buffer (98% formamide, 10 mM EDTA, 0.025% xylene cyanol FF, and 0.025% bromophenol blue), denatured for 3 min at 95°C, and analyzed on a 25% denaturing polyacrylamide gel containing 3 M urea in 1× TBE buffer or a 10% polyacrylamide gel containing 7 M urea in 1× TBE.

### Replication initiation assay

Each 25-μl reaction contained 25 mM tris-HCl (pH 8.0), 50 mM NaCl, BSA (100 μg/ml), 10 mM MgCl_2_, 10 mM DTT, 400 μM ATP, 150 μM GTP, 150 μM CTP, 15 μM UTP, 100 μM dATP, 100 μM dGTP, 100 μM ddCTP as indicated, 10 μM dTTP, 0.027 μM α-[^32^P]dTTP (3000 Ci/mmol), and 4 nM supercoiled pUC19 containing LSP (mtDNA 1 to 477). All reactions (unless otherwise stated) contained 200 nM TFAM, 60 nM TFB2M, 20 nM POLRMT, 20 nM D274A POLγA exo^−^, 40 nM POLγB, and 2 nM RNase H1. mtSSB wild type or mutants were added as indicated.

The reactions were incubated at 32°C for 30 min. In this experiment, we labeled the DNA and not the RNA to detect whether the RNA primers are used by POLγ to start DNA synthesis. This experiment was designed to determine whether mutant mtSSB variants could stimulate the initiation of DNA replication. The reactions were stopped, treated, and analyzed in the same way as the in vitro transcription assays but run on a 6% denaturing polyacrylamide gel containing 7 M urea in 1× TBE buffer or a 25% polyacrylamide gel containing 3 M urea in 1× TBE.

### Preparation of crude mitochondria and mitochondrial RNA

Isolation of mitochondria from mouse hearts was performed as previously described (*62*). HeLa cells were harvested at about 80% confluence and spun down at 500*g* for 3 min at 4°C and then washed once with ice-cold 1× PBS. The cell pellet was resuspended in 1 ml of lysis buffer [20 mM tris-HCl (pH 7.6), 220 mM mannitol, 70 mM sucrose, 1 mM EDTA, 1× protease inhibitor (1 mM phenylmethylsulfonyl fluoride, 2 μM pepstatin, 0.6 μM leupeptin, and 2 mM benzamidine), and BSA (100 μg/ml)] and incubated on ice for 15 min. The pellet was transferred to a glass Dounce homogenizer (1 ml), and the cells were broken by 20 to 25 strokes, followed by centrifugation at 1200*g* for 3 min at 4°C. The supernatant was transferred to an Eppendorf tube and spun at 14,000*g* for 3 min at 4°C to pellet the crude mitochondria. Mitochondrial RNA was prepared from crude mitochondria by TRIzol extraction, and after ethanol precipitation, the RNA was dissolved in nuclease-free water and treated with RNase-free DNase I at 37°C for 20 min, followed by purification with Quick-RNA Microprep column.

### Cappable-seq and analysis

Cappable-seq library preparation and sequencing were performed with RNA isolated from HeLa (human) or heart (mouse) mitochondria, and sequencing was performed by Vertis Biotechnologie (Freising, Germany). Single-end sequencing reads from Cappable-seq were trimmed of adapter sequences with cutadapt 1.18 (-a AGATCGGAAGAGCACACGT-CTGAACTCCAGTCAC –m 10) (*63*) and mapped to the human (GRCh38) or mouse (GRCm38) genome using Bowtie2 (*64*) with default parameters. 5′-end extraction and visualization were performed with samtools version 1.9 (*65*) and bedtools version 2.28.0 (*66*). TSS counts were normalized to total mitochondrial reads. Relative log_2_ fold changes across a 25-bp window were calculated using normalized TSS from biological replicates plus a pseudo count of one in HeLa cells (si*SSBP1* versus siCtrl) or in mouse heart (*L/L, cre* versus *L/L*) with bigwigCompare version 3.1.3 (--binSize 25) (*67*). Circos plot was generated with Circos version 0.93 (*68*). To obtain sequences surrounding nonspecific TSS, positions with more than a fourfold relative change were extracted with bedtools and a customized R script, and sequence logos were generated using Skylign (Information content – above background) (*69*).

### Transcriptome and small RNA-seq analysis

Total RNA was extracted form 5-week-old mouse heart tissues with a QIAGEN miRNeasy kit according to the kit instructions for transcriptome analysis. Mitochondrial RNA was extracted with the same kit from isolated heart mitochondria of 5-week-old mice for small RNA-seq analysis. For TruSeq, sequences reads were aligned to the mouse genome (GRCm38.p6 primary assembly, masked for nuclear mitochondrial sequences) using STAR version 2.7.3a (*70*) and the GENCODE vM24 gene annotation with a customized mitochondrial annotation. Gene expression quantification was performed with Salmon (-l ISR --seqBias) (*71*) in alignment-based mode on the transcriptome alignment produced by STAR using a transcriptome sequence file generated with gffread (*72*).

Differential expression analysis was performed with DESeq2 (*73*) using counts summarized by tximport (*74*), and effect size estimation was performed using apeglm (*75*). Full fragment length mitochondrial coverage profiles were generated with samtools version 1.10 (*65*) and bedtools version 2.26.0 (*66*) and normalized to total reads mapped to mitochondria, and circular figures were generated with Circos version 0.93 (*68*).

For small RNA-seq, sequence reads were trimmed of adapter sequences with BBDuk (ktrim = r, kink = 4, k = 18), and paired ends were merged with BBMerge (useoverlap = t pfilter = 1 mininsert = 18 mininsert0 = 18 trimonoverlapping = t). Merged reads were aligned to the mouse genome with Bowtie2 (*76*). Mitochondrial coverage profiles were generated with samtools and bedtools genomecov and normalized to total reads mapped to the mitochondrial genome, and circular figures were generated with Circos.

## SUPPLEMENTARY MATERIALS

Supplementary material for this article is available at http://advances.sciencemag.org/cgi/content/full/7/27/eabf8631/DC1

## Appendix

View/request a protocol for this paper from *Bio-protocol*.
